# Clinical and Biochemical Outcomes in Transgender Individuals Undergoing Hormone Therapy: Protocol for a Systematic Review

**DOI:** 10.2196/57931

**Published:** 2024-11-12

**Authors:** Emily Sattora, Karen Teelin, Christopher Prendergast, Abigail Smith, James Evans, Aamer Imdad

**Affiliations:** 1 Department of Public Health Norton College of Medicine SUNY Upstate Medical University Syracuse, NY United States; 2 Section of Adolescent Medicine SUNY Upstate Medical University Syracuse, NY United States; 3 Division of Pediatric Cardiology SUNY Upstate Medical University Syracuse, NY United States; 4 Health Sciences Library Upstate Medical University Syracuse, NY United States; 5 Division of Pediatric Gastroenterology, Hepatology, Pancreatology, and Nutrition Stead Family Department of Pediatrics University of Iowa Carver College of Medicine Iowa City, IA United States

**Keywords:** transgender, lipid levels, hormone therapy, biochemical outcomes, clinical outcomes, comprehensive data, systematic review, meta-analysis, adolescent, adults, electronic databases, testosterone, estrogen

## Abstract

**Background:**

Monitoring of various clinical outcomes and parameters, such as lipid levels, is recommended in transgender individuals undergoing hormone therapies. However, comprehensive data to inform these recommendations is scarce.

**Objective:**

This study aims to conduct a systematic review and meta-analysis to synthesize evidence from existing literature on the effect of exogenous hormone therapy on clinical and biochemical outcomes for transgender adolescents and adults.

**Methods:**

We will search multiple electronic databases and will include prospective and retrospective observational studies with and without a control group. The study population will include transgender individuals undergoing hormone therapy with testosterone or estrogen. Comparisons will include age-matched, cisgender individuals and changes from baseline. Primary outcomes include changes in or the development of abnormal lipid parameters. Secondary outcomes include BMI, weight, height, and blood pressure for age, serum testosterone or estrogen levels, and development of disease including hypertension, diabetes, fatty liver disease, obesity, adverse cardiac events, as well as all-cause mortality. The meta-analysis will pool the studies where applicable, and meta-regressions will be conducted to evaluate effect modifiers. The GRADE (Grading of Recommendations Assessment, Development, and Evaluation) approach will be used to evaluate the overall certainty of evidence.

**Results:**

We will summarize the selection of the eligible studies using a PRISMA (Preferred Reporting Items for Systematic Reviews and Meta-Analyses) flowchart. The results will be presented in a table summarizing the evidence. Data collection is ongoing, and the paper is expected to be published in Spring 2025.

**Conclusions:**

This systematic review will summarize and evaluate the evidence of the clinical and biochemical outcomes associated with hormone therapies for transgender individuals.

**Trial Registration:**

PROSPERO CRD42024483138; https://tinyurl.com/yc4sfvnb

**International Registered Report Identifier (IRRID):**

PRR1-10.2196/57931

## Introduction

An estimated 1.6 million adolescents and adults identify as transgender in the United States [1]. For those seeking hormone therapy as part of their gender-affirming care, the World Professional Association for Transgender Health (WPATH) recommends initiation and continuation of gender-affirming hormone therapy in eligible transgender and gender-diverse individuals who require treatment based on demonstrated improvement in quality of life and psychosocial functioning [2]. The Endocrine Society guidelines recommend a testosterone therapy regimen consisting of intramuscular or subcutaneous testosterone at an initial dose of 25 mg/m^2^ every 2 weeks with a gradual increase in the dose every 6 months until an adult dose of 100-200 mg every 2 weeks is achieved [3]. For transfeminine adolescents, the recommendation is an initial dose of 5 µg/kg per day of oral 17B-estradiol gradually increased every 6 months until an adult dose of 2-6 mg per day is achieved or an initial dose of 6.25-12.5 µg transdermally until an adult dose of 50-200 µg/24 hours is achieved [3].

**Figure 1:**
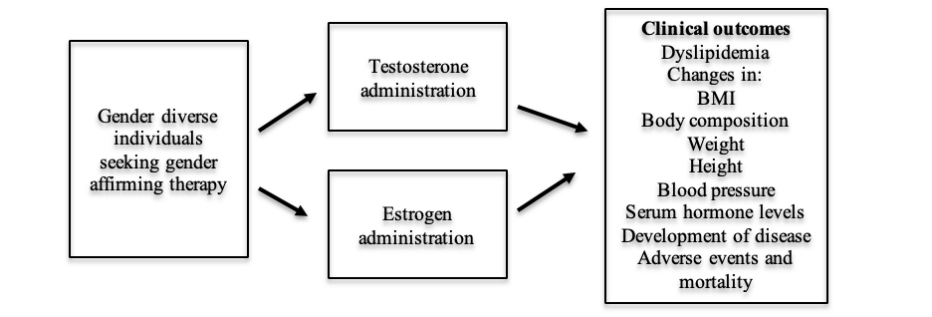
A conceptual framework for the association between estrogen and testosterone use and lipid metabolism.

Abnormal lipid levels have been associated with an increased risk of atherosclerosis and cardiovascular disease throughout life [4]. The pathophysiology of testosterone’s and estradiol’s effect on lipid metabolism is complex and has remained largely unknown [5,6]. However, recent studies have examined the relationship between sex hormones and lipid metabolism in an attempt to establish causation. A review published by Zhang et al [7] in 2022 postulates that in human adipocyte cells, estrogen increases the expression of certain enzymes that inhibit lipogenesis. A different study from 2022 showed a similar relationship between testosterone and lipid metabolism, stating that low testosterone leads to major changes across various metabolic functions [8]. Despite these findings, the extent of the association between testosterone and estrogen on lipid metabolism remains largely unknown. Figure 1 shows a conceptual framework for the association between these hormones and lipid metabolism. Currently, treatment guidelines recommend monitoring lipid levels in those taking gender-affirming testosterone therapy. However, the recommended frequency of such monitoring is unclear [2,3]. Monitoring of lipid levels is not currently recommended for those taking gender-affirming estrogen therapy, despite inconsistencies in existing literature [9]. The Endocrine Society reports a wide variety of possible adverse effects when undergoing testosterone therapy, including coronary artery disease and hypertension [3]. Individuals undergoing estrogen therapy face similar potential adverse outcomes, including the development of thromboembolic disease, coronary artery disease, and hypertriglyceridemia [3].

There has been a recent increase in research investigating the effects of gender-affirming testosterone therapy on lipid levels in transgender and gender-diverse adolescents and adults. A recent systematic review and meta-analysis published by Tienforti et al [10] reviewed 39 existing studies. It concluded that testosterone-based gender-affirming hormone therapy in individuals assigned female at birth was statistically associated with changes in body composition, body fat distribution, and the cardiovascular lipid profile. This study described the results comprehensively; however, it did not use any method that can incorporate the risk of bias into the conclusion, such as methods described by the GRADE (Grading of Recommendations, Assessment, Development, and Evaluation) working group. Furthermore, this systematic review excluded studies that compare the clinical and biochemical outcomes for age-matched, cisgender females. Our study seeks to describe the totality of evidence using the GRADE methods and includes studies that make use of an age-matched, cisgender female population. In addition, we plan to include studies that discuss clinical outcomes in transfeminine adolescents undergoing estrogen hormone therapy matched with similarly aged cisgender males. We also plan to do additional analyses, such as evaluating associations of age with clinical and biochemical outcomes, with separate analyses for adolescent and adult populations. Separate analyses for these populations are necessary, given the differences in reference lipid values seen in adult versus adolescent populations [3,11].

## Methods

### Objective

To assess the effects of testosterone and estrogen therapies on select clinical and biochemical outcomes of transgender and gender-diverse adolescents and adults.

### Inclusion Criteria

#### Study Type

We will include prospective and retrospective observational cohort studies with and without a control group. We will exclude case-control studies, reviews, case reports, cross-sectional studies, and qualitative studies.

#### Population

Our population will include transmasculine and transfeminine individuals taking gender-affirming testosterone or estrogen therapy. We will include all individuals irrespective of age.

#### Intervention and Exposure

Our primary exposure is exogenous testosterone or estrogen hormone therapy, which will be categorized as yes or no (any exposure to hormone therapy for at least one year). We will record the duration of exposure, and studies that include a duration of therapy of less than 6 months will be excluded. We will include studies of multiple methods of exogenous hormone administration, including subcutaneous, transdermal, and intramuscular routes for testosterone, as well as oral, transdermal, and intramuscular routes for estrogen. Subcutaneous injections are administered under the skin, often in the abdomen or thigh, typically with a 5/8-inch needle [12]. Intramuscular injections are administered into the muscle, frequently the thigh muscle, with a larger needle (often 1 inch) [12]. Transdermal routes include patches that may be applied to the back, abdomen, stomach, thighs, or arms [13]. Transdermal routes of administration may also incorporate solutions or gels [13]. We will exclude studies where testosterone or estrogen were administered for reasons aside from gender-affirming care.

#### Comparison

We will include studies that include age-matched cisgender populations as comparisons, but studies that follow individual changes from baseline data will be included as well. Any study that allows for the determination of an association between the exposure and a relevant outcome will be considered for inclusion, regardless of the presence of absence of a control group (Textbox 1).

**Inclusion and exclusion criteria.**

**Inclusion criteria:**

- Study type: prospective and retrospective observational studies.
- Population: transmasculine and transfeminine individuals of all ages.
- Intervention (exposure): testosterone or estrogen therapy administered transdermally, intramuscularly, subcutaneously, or orally for >6 months.
- Comparison: age-matched cisgender populations or changes from baseline.

**Exclusion criteria:**

- Study type: case-control, reviews, case reports, cross-sectional, and qualitative studies.
- Intervention (exposure): therapy administered for reasons aside from gender-affirming therapy or for a mean duration of <6 months.

#### Outcomes

The primary outcome of interest will be changes in lipid levels. Secondary outcomes of interest include a variety of clinical and biochemical variables. Values will be measured at baseline, 1 year, and longest follow-up. Studies will be included if they report data for any of the primary or secondary outcomes. Studies need not report every potential outcome to be included in the analysis. The following values will be included for consideration (Textbox 2).

Lipid levels will be compared with reference ranges for healthy adolescents and adults (more details in Tables 1 and 2). Reference ranges were adapted from the NIH and Cleveland Clinic’s guidelines for cardiovascular health [3,11]. Lipid levels will be measured at baseline, one year, and longest follow-up. Any values outside of the acceptable ranges shown in Tables 1 and 2 will be considered abnormal. We will assess mean differences and mean changes from baseline levels and include relative and absolute risk measures when relevant.

Each lipid parameter will be evaluated as a dichotomous outcome (abnormal, yes or no), and the mean difference and mean changes from baseline will be continuously assessed. Any values outside of the acceptable normal ranges given in the above tables will be considered an abnormal outcome.

**The primary and secondary outcomes.**

Continuous outcome:

- Total cholesterol (TC), a measure of the cumulative amount of cholesterol in the blood [14].
- Low-density lipoprotein (LDL), often referred to as “bad” cholesterol, or the main source of buildup in the arteries [14].
- High-density lipoprotein (HDL), referred to as “good” cholesterol. HDL helps in the removal of other forms of cholesterol from the bloodstream [14].
- Non-HDL cholesterol (non-HDL), a measure of the total cholesterol minus the HDLs [14].
- Triglycerides (TGs), the most common type of fat found in the body [14].
- BMI for age (kg/m^2^ or *z* scores).
- Body composition (visceral fat, body fat, bone density, and muscle mass).
- Weight for age (kg or *z* scores).
- Height for age (cm or *z* scores).
- Blood pressure for age (mmHg).
- Serum testosterone levels (ng/dL or nmol/L).
- Serum estrogen levels (pg/mL).

Dichotomous outcome:

- Development of hypertension.
- Development of type-2 diabetes.
- Development of fatty liver disease.
- Development of obesity (Defined by age and sex-specific BMI in the 95th percentile or greater for adolescents [15], or a BMI of 30 or higher for adults older than 20 years) [16].
- Presence or absence of any adverse cardiac advents.
- All-cause mortality.

**Table 1:** Acceptable lipid parameter ranges in cisgender adolescents aged 10-19 years [3].

Category	Acceptable range (mg/dL)
TC^a^	<170
LDL^b^	<110
Non-HDL^c^	<120
HDL^d^	>45
TG^e^	<90

^a^TC: total cholesterol.

^b^LDL: low-density lipoprotein.

^c^Non-HDL: non–high-density lipoprotein.

^d^HDL: high-density lipoprotein.

^e^TG: triglycerides.

**Table 2:** Acceptable lipid parameter ranges in cisgender adults [11].

Category	Acceptable range (mg/dL)
TC^a^	125-200
LDL^b^	<100
Non-HDL^c^	<130
HDL^d^	>40 (Males) >50 (females)
TG^e^	<150

^a^TC: total cholesterol.

^b^LDL: low-density lipoprotein.

^c^Non-HDL: non–high-density lipoprotein.

^d^HDL: high-density lipoprotein.

^e^TG: triglycerides.

### Literature Search

Systematic searches will be conducted electronically on several databases, including PubMed, Scopus, Embase, Web of Science, the Cochrane Central Register for Controlled Trials, the World Health Organization Global Index Medicus, and CINHAL. We will also examine the references of formerly published reviews for possible inclusion. The citation tracking function of included studies through PubMed will be reviewed for eligible studies. ClinicalTrials.gov will be used to identify any studies that are currently ongoing. No restrictions will be applied to initial searches based on study design, publication date or status, language, or outcomes. We will include studies published to date. A full search strategy, including search terms, can be found in Multimedia Appendix 1.

### Data Extraction and Synthesis

#### Selection of Studies

A 3-stage process will be used to screen studies for inclusion. To begin, 2 individuals will screen study titles and abstracts yielded from the systematic searches independently. Any studies selected during this stage will advance to a full-text review, which constitutes the second stage. Finally, eligible studies will undergo data extraction during the final stage. A coding software, Covidence (Veritas Health Innovation), will be used to assist with screening and data extraction [17]. Any conflicts that arise during the study selection process will be resolved with discussion. For any studies that are only available in abstract form, we will write to the authors or use the interlibrary loan system to obtain the full text. If any included studies are available in a language other than English, we will use local resources for translation. Multiple publications of the same study will be counted as one, and data will be extracted from all available sources as needed.

#### Data Extraction

A data extraction sheet will be designed and used to collect information on selected studies. Similar to screening methods, 2 individuals will use the data extraction sheet independently, followed by a comparison of their findings. Any conflicts or questions will be resolved by discussion, and a senior author will be involved if necessary. For each study, the following data will be extracted: type of study, study site and year, study population characteristics including mean age, exposure (exogenous sex hormone therapy, including route of administration, dose, duration, and frequency), comparisons, primary and secondary outcomes, confounding factors, and risk of bias. Confounding factors that will be considered include sex assigned at birth, ethnicity, age, and familial history, as these variables may all play a role in lipid metabolism [11,18]. If both adjusted and unadjusted values are available, we will use the adjusted values for analysis.

#### Assessment of Risk of Bias in Included Studies

The Cochrane risk of bias in non-randomized studies (ROBINS-1) tool will be used to assess the risk of bias in included studies [19]. A total of 2 authors will assess the risk of bias independently before coming to a consensus. Any disagreements will be resolved by discussion, with a senior author consulting if no agreement can be reached. In addition, 5 domains of signaling questions will be addressed, including bias in the selection of participants, bias due to confounding, bias due to missing data, bias in selecting the reported result, and bias due to measurement of outcomes. Each domain will be judged individually and will be awarded a label of low, moderate, or critical risk of bias. The overall risk of bias will be determined from the highest risk label across the domains.

#### Data Synthesis

Findings from this systematic review will be reported qualitatively and quantitatively. Qualitatively, we will use a narrative synthesis to report our results and the characteristics of the included studies. Quantitively, meta-analyses will be conducted to synthesize data across studies so long as clinical and methodological homogeneity exists. Relative risk effect sizes, along with their 95% CIs, will be used to measure dichotomous outcomes. Mean difference effect sizes and their corresponding 95% CIs will be used to measure change from baseline for continuous outcomes. The mean difference will be calculated using the absolute difference between the mean values between the 2 groups in the study. If the study contains a cisgender comparison group, mean differences will be calculated between the intervention and control groups. If the study does not contain a cisgender comparison group, the mean difference will be calculated using the pre- and post-values from the intervention group. Data from these 2 study types will be analyzed separately throughout the analysis, including in measures of relative and absolute risk. Random effect models will be used to pool data using the generic inverse variance method of meta-analysis. Data from studies that measured change over time will be pooled along with data matched to cisgender controls. Studies that make use of multiple intervention groups will be combined and analyzed as a single study. In addition, a meta-regression will be conducted to isolate effect modifiers and evaluate differential effects based on factors such as mean age of study (continuous outcome), duration of exposure in days, and route of administration. To attain these statistics, we will make use of RevMan (Cochrane) [20] and STATA software (StataCorp LLC) [21].

#### Dealing With Missing Data

Attrition will be documented during the data extraction process. We will contact the trial authors to request full datasets if data is missing for some cases. If the SDs are not reported for continuous outcomes, we will request this data from the study authors. In the event of a nonresponse or unavailability, we will attempt to use an SD value from a similar study with a similar population. In the event that final values are not available but values are given for the difference between the study start and end, we will use the difference to calculate the final values.

### Assessment of Heterogeneity

Heterogeneity, or any variability among studies, can be assessed with measures of clinical, methodological, or statistical heterogeneity. We will analyze the statistical heterogeneity of effect sizes in the total data by using statistics such as Tau^2^, chi-square, and *I*^2^. We will also visually inspect forest plots and assess *P* values. Significant statistical heterogeneity will be achieved at *P*<.10 and an *I*^2^ value exceeding 50%. We would also expect our forest plots to show substantial variability in the effect of the intervention. In addition, subgroup analysis will be conducted to determine the reasoning behind any identified statistical heterogeneity. These subgroups may assess differences in study populations, exposure routes, and outcomes.

### Assessment of Reporting Bias

Biases resulting from small study size or publication bias will be evaluated using funnel plots. Given an asymmetrical funnel plot, weighted linear regression (Egger) tests will be used to determine the presence of bias when at least 10 studies are included in the meta-analysis.

### Subgroup Analysis

Subgroup analysis will be done by age group (adolescents <19 years of age vs adults ≥19 years of age), route of exposure, and duration of exposure (>2 years vs <2 years).

### Sensitivity Analysis

We will remove studies with a high risk of bias in order to complete sensitivity analysis. Results will be compared between random effects and fixed-effect models.

### Rating of Overall Quality of Evidence

The GRADE approach will be used to evaluate the overall quality of evidence [22]. The GRADE approach uses characteristics such as study design, risk of bias, heterogeneity of effect, directness of evidence, publication bias, and precision of effect estimates to assess the overall quality of evidence for an outcome [22]. The GRADEpro software (GRADE Working Group) [23] will be used to rate the overall quality of evidence as very low, low, moderate, or high. The GRADE assessment results will be presented in a summary of findings table that will include quality ratings for the primary and secondary outcomes of total cholesterol, LDL, HDL, obesity, hypertension, and quality of life scores.

## Results

The results of this review intend to provide insights into various clinical and biochemical outcomes that result from the use of hormone therapies in transgender individuals. The results will undergo peer review and be submitted for publication. Data collection is ongoing and the review is expected to be published in spring 2025.

## Discussion

The protocol for this systematic review describes a comprehensive approach to a review of the literature in order to draw meaningful conclusions. It is our hope that this review will serve to inform future clinical guidelines and practices for this marginalized group. The strengths of this review include the use of 2 authors for screening for all papers, the inclusion of literature from multiple electronic databases, and the inclusion of a wide variety of outcomes. In addition, GRADE criteria will enable us to assess the overall strength and quality of evidence. It is possible that all included studies will not report data for all the outcomes of this review, which is a limitation of this study.

Even though we defined the methods of data extraction and analysis for this systematic review a priori, data may not be available for all outcomes in all included studies, which would be a limitation of this systematic review. An ideal comparison to assess the effect of hormone therapy in transgender individuals includes a cisgender without the hormone supplements. However, studies may not include a cisgender comparison group, and in that case, we will consider the analysis for pre- and post-hormone supplementation in transgender individuals. We, however, plan to analyze the data separately for cisgender comparison and pre-post intervention data in the same individual.

## Appendix

Multimedia Appendix 1 Search strategy.

## Abbreviations

GRADE: Grading of Recommendations Assessment, Development, and Evaluation
PRISMA: Preferred Reporting Items for Systematic Reviews and Meta-Analyses
ROBINS-1: risk of bias in non-randomized studies
WPATH: World Professional Association for Transgender Health

## Data Availability

The datasets generated during and/or analyzed during this study are available from the corresponding author on reasonable request.
