# Supplementary material for: Clinical and Biochemical Outcomes in Transgender Individuals Undergoing Hormone Therapy: Protocol for a Systematic Review
Source: JMIR Res Protoc. 2024 Nov 12;13:e57931. doi: 10.2196/57931 (PMC11599881; doi:10.2196/57931)
Supplement: Multimedia Appendix 1 [file resprot_v13i1e57931_app1.pdf]

Run Date February 7th, 2024

PubMed: 121 Results

("Transgender Persons"[MeSH Terms] OR ("transgender*"[Title/Abstract] OR "trans people"[Title/Abstract] OR "trans person*"[Title/Abstract] OR "transpeople"[Title/Abstract] OR "transperson*"[Title/Abstract] OR "trans man"[Title/Abstract] OR "trans men"[Title/Abstract] OR "trans male*"[Title/Abstract] OR "transmale*"[Title/Abstract] OR "trans masculin*"[Title/Abstract] OR "transmasculin*"[Title/Abstract] OR "transman"[Title/Abstract] OR "transmen"[Title/Abstract] OR "trans woman"[Title/Abstract] OR "trans women"[Title/Abstract] OR "transwoman"[Title/Abstract] OR "transwomen"[Title/Abstract] OR "trans female*"[Title/Abstract] OR "transfemale*"[Title/Abstract] OR "trans feminin*"[Title/Abstract] OR "transfeminine*"[Title/Abstract] OR "transexual*"[Title/Abstract] OR "transsexual*"[Title/Abstract])) AND ("Hormone Replacement Therapy"[MeSH Terms] OR "gonadal steroid hormones/therapeutic use"[MeSH Terms] OR ("hormone replacement*"[Title/Abstract] OR "hormone therap*"[Title/Abstract] OR "hormonal therap*"[Title/Abstract] OR "hormone treatment*"[Title/Abstract] OR "endocrine therap*"[Title/Abstract] OR "endocrine treatment*"[Title/Abstract] OR "endocrinotherapy"[Title/Abstract] OR "estrogen therap*"[Title/Abstract] OR "estrogen treatment*"[Title/Abstract] OR "estrogen replacement*"[Title/Abstract] OR "estrogen substitution*"[Title/Abstract] OR "oestrogen therap*"[Title/Abstract] OR "oestrogen treatment*"[Title/Abstract] OR "oestrogen replacement*"[Title/Abstract] OR "oestrogen substitution*"[Title/Abstract] OR "testosterone therap*"[Title/Abstract] OR "testosterone treatment*"[Title/Abstract] OR "testosterone replacement*"[Title/Abstract] OR "testosterone substitution*"[Title/Abstract] OR "androgen therap*"[Title/Abstract] OR "androgen treatment*"[Title/Abstract] OR "androgen replacement*"[Title/Abstract] OR "androgen substitution*"[Title/Abstract])) AND ("Cholesterol"[MeSH Terms] OR "Triglycerides"[MeSH Terms] OR ("lipid*"[Title/Abstract] OR "cholesterol*"[Title/Abstract] OR "cholesterin*"[Title/Abstract] OR "high density lipoprotein*"[Title/Abstract] OR "HDL"[Title/Abstract] OR "low density lipoprotein*"[Title/Abstract] OR "LDL"[Title/Abstract] OR "triglyceride*"[Title/Abstract] OR "triacylglycerol*"[Title/Abstract]))

Embase: 243 results

#1 ‘transgender’/exp OR ‘transgender*’:ti,ab OR ‘trans people’:ti,ab OR ‘trans person*’:ti,ab OR ‘transpeople’:ti,ab OR ‘transperson*’:ti,ab OR ‘trans man’:ti,ab OR ‘trans men’:ti,ab OR ‘trans male*’:ti,ab OR ‘transmale*’:ti,ab OR ‘trans masculin*’:ti,ab OR ‘transmasculin*’:ti,ab OR ‘transman’:ti,ab OR ‘transmen’:ti,ab OR ‘trans woman’:ti,ab OR ‘trans women’:ti,ab OR ‘transwoman’:ti,ab OR ‘transwomen’:ti,ab OR ‘trans female*’:ti,ab OR ‘transfemale*’:ti,ab OR ‘trans feminin*’:ti,ab OR ‘transfeminine*’:ti,ab OR ‘transexual*’:ti,ab OR ‘transsexual*’:ti,ab

#2 ‘hormone substitution’/exp OR ‘hormone replacement therap*’:ti,ab OR ‘hormone replacement*’:ti,ab OR ‘hormone therap*’:ti,ab OR ‘hormonal therap*’:ti,ab OR ‘hormone treatment*’:ti,ab OR ‘endocrine therap*’:ti,ab OR ‘endocrine treatment*’:ti,ab OR ‘endocrinotherapy’:ti,ab OR ‘estrogen therap*’:ti,ab OR ‘estrogen treatment*’:ti,ab OR ‘estrogen replacement*’:ti,ab OR ‘estrogen substitution*’:ti,ab OR ‘oestrogen therap*’:ti,ab OR ‘oestrogen treatment*’:ti,ab OR ‘oestrogen replacement*’:ti,ab OR ‘oestrogen substitution*’:ti,ab OR ‘testosterone therap*’:ti,ab OR ‘testosterone treatment*’:ti,ab OR ‘testosterone replacement*’:ti,ab OR ‘testosterone substitution*’:ti,ab OR ‘androgen therap*’:ti,ab OR ‘androgen treatment*’:ti,ab OR ‘androgen replacement*’:ti,ab OR ‘androgen substitution*’:ti,ab

#3 'cholesterol'/exp OR 'triacylglycerol'/exp OR ‘lipid*’:ti,ab OR ‘cholesterol*’:ti,ab OR ‘cholesterin*’:ti,ab OR ‘high density lipoprotein*’:ti,ab OR ‘HDL’:ti,ab OR ‘low density lipoprotein*’:ti,ab OR ‘LDL’:ti,ab OR ‘triglyceride*’:ti,ab OR ‘triacylglycerol*’:ti,ab

#4 #1 AND #2 AND #3

CINAHL: 38 results

S14 S4 AND S8 AND S13
S13 S9 OR S10 OR S11 OR S12
S12 AB (“lipid*” OR “cholesterol*” OR “cholesterin*” OR “high density lipoprotein*” OR “HDL” OR “low density lipoprotein*” OR “LDL” OR “triglyceride*” OR “triacylglycerol*”)
S11 TI (“lipid*” OR “cholesterol*” OR “cholesterin*” OR “high density lipoprotein*” OR “HDL” OR “low density lipoprotein*” OR “LDL” OR “triglyceride*” OR “triacylglycerol*”)
S10 (MH "Triglycerides")
S9 (MH "Cholesterol")
S8 S5 OR S6 OR S7
S7 AB (“hormone replacement therap*” OR “hormone replacement*” OR “hormone therap*” OR “hormonal therap*” OR “hormone treatment*” OR “endocrine therap*” OR “endocrine treatment*” OR “endocrinotherapy” OR “estrogen therap*” OR “estrogen treatment*” OR “estrogen replacement*” OR “estrogen substitution*” OR “oestrogen therap*” OR “oestrogen treatment*” OR “oestrogen replacement*” OR “oestrogen substitution*” OR “testosterone therap*” OR “testosterone treatment*” OR “testosterone replacement*” OR “testosterone substitution*” OR “androgen therap*” OR “androgen treatment*” OR “androgen replacement*” OR “androgen substitution*”)
S6 TI (“hormone replacement therap*” OR “hormone replacement*” OR “hormone therap*” OR “hormonal therap*” OR “hormone treatment*” OR “endocrine therap*” OR “endocrine treatment*” OR “endocrinotherapy” OR “estrogen therap*” OR “estrogen treatment*” OR “estrogen replacement*” OR “estrogen substitution*” OR “oestrogen therap*” OR “oestrogen treatment*” OR “oestrogen replacement*” OR “oestrogen substitution*” OR “testosterone therap*” OR “testosterone treatment*” OR “testosterone replacement*” OR “testosterone substitution*” OR “androgen therap*” OR “androgen treatment*” OR “androgen replacement*” OR “androgen substitution*”)
S5 (MH "Hormone Replacement Therapy")
S4 S1 OR S2 OR S3

S3 AB (“transgender*” OR “trans people” OR “trans person*” OR “transpeople” OR “transperson*” OR “trans man” OR “trans men” OR “trans male*” OR “transmale*” OR “trans masculin*” OR “transmasculin*” OR “transman” OR “transmen” OR “trans woman” OR “trans women” OR “transwoman” OR “transwomen” OR “trans female*” OR “transfemale*” OR “trans feminin*” OR “transfeminine*” OR “transexual*” OR “transsexual*”)
S2 TI (“transgender*” OR “trans people” OR “trans person*” OR “transpeople” OR “transperson*” OR “trans man” OR “trans men” OR “trans male*” OR “transmale*” OR “trans masculin*” OR “transmasculin*” OR “transman” OR “transmen” OR “trans woman” OR “trans women” OR “transwoman” OR “transwomen” OR “trans female*” OR “transfemale*” OR “trans feminin*” OR “transfeminine*” OR “transexual*” OR “transsexual*”)
S1 (MH "Transgender Persons")

Scopus: 215 results

(TITLE-ABS-KEY(transgender* OR {trans people} OR "trans person*" OR transpeople OR transperson* OR {trans man} OR {trans men} OR "trans male*" OR transmale* OR "trans masculin*" OR transmasculin* OR transman OR transmen OR {trans woman} OR {trans women} OR transwoman OR transwomen OR "trans female*" OR transfemale* OR "trans feminin*" OR transfeminine* OR transexual* OR transsexual*)) AND (TITLE-ABS-KEY("hormone replacement*" OR "hormone therap*" OR "hormonal therap*" OR "hormone treatment*" OR "endocrine therap*" OR "endocrine treatment*" OR endocrinotherapy OR "estrogen therap*" OR "estrogen treatment*" OR "estrogen replacement*" OR "estrogen substitution*" OR "oestrogen therap*" OR "oestrogen treatment*" OR "oestrogen replacement*" OR "oestrogen substitution*" OR "testosterone therap*" OR "testosterone treatment*" OR "testosterone replacement*" OR "testosterone substitution*" OR "androgen therap*" OR "androgen treatment*" OR "androgen replacement*" OR "androgen substitution*")) AND (TITLE-ABS-KEY(lipid* OR cholesterol* OR cholesterin* OR "high density lipoprotein*" OR HDL OR "low density lipoprotein*" OR LDL OR triglyceride* OR triacylglycerol*))

CENTRAL: 11 results

#1 MeSH descriptor: [Transgender Persons] explode all trees

#2 (transgender* OR trans NEXT people OR trans NEXT person* OR transpeople OR transperson* OR trans NEXT man OR trans NEXT men OR trans NEXT male* OR transmale* OR trans NEXT masculin* OR transmasculin* OR transman OR transmen OR trans NEXT woman OR trans NEXT women OR transwoman OR transwomen OR trans NEXT female* OR transfemale* OR trans NEXT feminin* OR transfeminine* OR transexual* OR transsexual*):ti,ab

#3 #1 OR #2

#4 MeSH descriptor: [Hormone Replacement Therapy] explode all trees

#5 MeSH descriptor: [Gonadal Steroid Hormones] explode all trees and with qualifier(s): [therapeutic use - TU]

#6 (hormone NEXT replacement* OR hormone NEXT therap* OR hormonal NEXT therap* OR hormone NEXT treatment* OR endocrine NEXT therap* OR endocrine NEXT treatment* OR endocrinotherapy OR estrogen NEXT therap* OR estrogen NEXT treatment* OR estrogen NEXT replacement* OR estrogen NEXT substitution* OR oestrogen NEXT therap* OR oestrogen NEXT treatment* OR oestrogen NEXT replacement* OR oestrogen NEXT substitution* OR testosterone NEXT therap* OR testosterone NEXT treatment* OR testosterone NEXT replacement* OR testosterone NEXT substitution* OR androgen NEXT therap* OR androgen NEXT treatment* OR androgen NEXT replacement* OR androgen NEXT substitution*):ti,ab

#7 #4 OR #5 OR #6

#8 MeSH descriptor: [Cholesterol] explode all trees

#9 MeSH descriptor: [Triglycerides] explode all trees

#10 (lipid* OR cholesterol* OR cholesterin* OR high NEXT density NEXT lipoprotein* OR HDL OR low NEXT density NEXT lipoprotein* OR LDL OR triglyceride* OR triacylglycerol*):ti,ab

#11 #8 OR #9 OR #10

#12 #3 AND #7 AND #11

Web of Science: 121 results

#4 #1 AND #2 AND #3

#3 TS=(lipid* OR cholesterol* OR cholesterin* OR "high density lipoprotein*" OR HDL OR "low density lipoprotein*" OR LDL OR triglyceride* OR triacylglycerol*)

#2 TS=("hormone replacement*" OR "hormone therap*" OR "hormonal therap*" OR "hormone treatment*" OR "endocrine therap*" OR "endocrine treatment*" OR endocrinotherapy OR "estrogen therap*" OR "estrogen treatment*" OR "estrogen replacement*" OR "estrogen substitution*" OR "oestrogen therap*" OR "oestrogen treatment*" OR "oestrogen replacement*" OR "oestrogen substitution*" OR "testosterone therap*" OR "testosterone treatment*" OR "testosterone replacement*" OR "testosterone substitution*" OR "androgen therap*" OR "androgen treatment*" OR "androgen replacement*" OR "androgen substitution*")

#1 TS=(transgender* OR "trans people" OR "trans person*" OR transpeople OR transperson* OR "trans man" OR "trans men" OR "trans male*" OR transmale* OR

"trans masculin*" OR transmasculin* OR transman OR transmen OR "trans woman" OR "trans women" OR transwoman OR transwomen OR "trans female*" OR transfemale* OR "trans feminin*" OR transfeminine* OR transexual* OR transsexual*)

Global Index Medicus (AIM, IMEMR, IMSEAR, LILACS, WPRO): 2 Results

(tw:(transgender* OR "trans people" OR "trans person*" OR transpeople OR transperson* OR "trans man" OR "trans men" OR "trans male*" OR transmale* OR "trans masculin*" OR transmasculin* OR transman OR transmen OR "trans woman" OR "trans women" OR transwoman OR transwomen OR "trans female*" OR transfemale* OR "trans feminin*" OR transfeminine* OR transexual* OR transsexual*)) AND (tw:("hormone replacement*" OR "hormone therap*" OR "hormonal therap*" OR "hormone treatment*" OR "endocrine therap*" OR "endocrine treatment*" OR endocrinotherapy OR "estrogen therap*" OR "estrogen treatment*" OR "estrogen replacement*" OR "estrogen substitution*" OR "oestrogen therap*" OR "oestrogen treatment*" OR "oestrogen replacement*" OR "oestrogen substitution*" OR "testosterone therap*" OR "testosterone treatment*" OR "testosterone replacement*" OR "testosterone substitution*" OR "androgen therap*" OR "androgen treatment*" OR "androgen replacement*" OR "androgen substitution*")) AND (tw:(lipid* OR cholesterol* OR cholesterin* OR "high density lipoprotein*" OR HDL OR "low density lipoprotein*" OR LDL OR triglyceride* OR triacylglycerol*))
